# Multicomponent Droplet Evaporation on Chemical Micro-Patterned Surfaces

**DOI:** 10.1038/srep41897

**Published:** 2017-02-03

**Authors:** Minghao He, Dong Liao, Huihe Qiu

**Affiliations:** 1 Department of Mechanical and Aerospace Engineering, The Hong Kong University of Science and Technology, Clear Water Bay, Kowloon, Hong Kong SAR, China

## Abstract

The evaporation and dynamics of a multicomponent droplet on a heated chemical patterned surface were presented. Comparing to the evaporation process of a multicomponent droplet on a homogenous surface, it is found that the chemical patterned surface can not only enhance evaporation by elongating the contact line, but also change the evaporation process from three regimes for the homogenous surface including constant contact line (CCL) regime, constant contact angle (CCA) regime and mix mode (MM) to two regimes, i.e. constant contact line (CCL) and moving contact line (MCL) regimes. The mechanism of contact line stepwise movement in MCL regimes in the microscopic range is investigated in detail. In addition, an improved local force model on the contact line was employed for analyzing the critical receding contact angles on homogenous and patterned surfaces. The analysis results agree well for both surfaces, and confirm that the transition from CCL to MCL regimes indicated droplet composition changes from multicomponent to monocomponent, providing an important metric to predict and control the dynamic behavior and composition of a multicomponent droplet using a patterned surface.

Droplet evaporation is a fundamental phenomenon in nature and has implications in different industries and research areas, from macroscale to nanoscale: thermal management^1^,^2^, fuel preparation^3^, inkjet printing^4^, microelectromechanical systems (MEMS) fabrication^5^, biosensing^6^ and DNA spotting^7^. Driven by such a wide range of applications, this phenomenon has been intensively studied for decades^8^,^9^,^10^,^11^. Compared to a monocomponent droplet, evaporation of a multicomponent droplet is more practical and common but has a more complicated mechanism. Thus, many researchers have been attracted to this area and efforts have been made to understand the underlying physical mechanisms of multicomponent droplet evaporation utilizing experimental and numerical methods.

Among the earliest researchers of numerical study for multicomponent droplets, Newbold and Amundson^12^ derived a model for describing the evaporation of a multicomponent droplet near its boiling point. Later, many models based on different approaches, such as expanded diffusion limit model^13^,^14^,^15^, linear stability approach^16^, continuous distribution model^17^ and continuous thermodynamics model^18^, have been built up in order to describe and predict multicomponent droplet evaporation. Meanwhile, experimental studies have been carried out under different environmental conditions to understand the mechanism of multicomponent evaporation, including suspending in an electric field^19^, free falling in a temperature gradient field^20^, sitting on heated or non-heated flat surface^21^,^22^, evaporating in heated air flow^23^ and at elevated pressures and temperatures^24^. Birdi and Vu^25^ studied the effect of surface wettability on droplet evaporation. Their results show that droplet evaporation on a Teflon surface (Hydrophobic surface) has a lower evaporation rate than on a glass surface (hydrophilic surface). A recent study by Dash and Garimella^26^ also shows that the higher the surface hydrophobicity is, the lower the droplet evaporation rate will be.

However, many investigations of multicomponent droplet evaporation, numerically or experimentally, were carried out on homogenous substrates but rarely related to heterogeneous surfaces. It is well known that heterogeneous surfaces, such as micro/nano structured^27^,^28^,^29^ and chemical patterned surface^30^, are able to control evaporation and the dynamic characteristics of monocomponent droplets^31^,^32^ and have promising applications such as anti-icing^33^, self-cleaning^31^ and droplet transportation^30^. Therefore, although the mechanism is still unknown, it is possible that the heterogeneous surface can control the evaporation and dynamic characteristics of a multicomponent droplet. Besides, from our previous experimental studies^34^,^35^ and numerical study of previous researchers^36^,^37^,^38^ in pool boiling, it is also shown that the chemical patterned surface can enhance the heat transfer efficiency by enhancing the phase change. Recently, molecular dynamic simulation results^39^ showed that the nanoscale chemical patterned surface could enhance the evaporation rate of water film, which implied the possibilities to enhance and control evaporation of multicomponent droplets on a patterned surface. Therefore, it is necessary to understand the mechanism of a multicomponent droplet on a structured surface, especially a chemical patterned surface.

Motivated by the possibilities mentioned above, the aim of this work is to perform a systematic experimental study of multicomponent (water-ethanol mixtures) droplets with different composition (0 to 15 ethanol vol.%) evaporating on a heated chemical patterned surface with a hydrophilic substrate and hydrophobic islands, compared to a homogeneous hydrophilic surface. We investigate and compare the evaporation characteristics of multicomponent droplets on those two surfaces, including evaporation duration, evaporation process, contact angle and base diameter. Evaporation enhancement introduced by a patterned surface is confirmed and evaporation processes on different surfaces, which can be divided in different regimes, are analyzed. Besides, the macro- and microscopic contact line movement have been revealed in the present work, in order to understand how the patterned surface controls contact line during evaporation. Furthermore, dynamic contact angle, especially the receding contact which indicates a change of evaporation regimes and is important to evaporation dynamic control, is studied in detail. Analysis of the critical receding contact angle was carried out with improved local forces model for the contact line, which gives a good explanation to the changes of the receding contact angle on both surfaces. This study investigated the mechanism and provides a new understanding of the influence of a patterned surface on multicomponent droplet evaporation, which will be important to surface design and droplet control in industrial applications.

## Results

### Chemical micro-patterned surface

**Figure 1:**
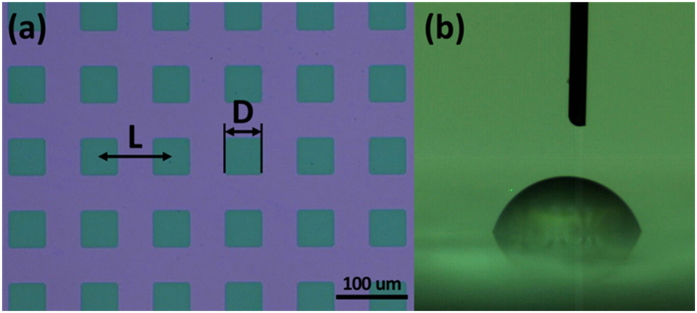
Microscopy image of patterned surface (**a**) with square Teflon patterns (green) of side length D = 50 μm and pattern-to-pattern spacing L of 100 μm and (**b**) typical recording image during evaporation.

Utilizing photolithography and plasma dry etching method (see Method and Supplementary Figure S3), the fine chemical micro-patterned surface was fabricated. Compared to the typical lift-off method, patterns defined by dry etching can achieve higher resolution. The patterned surface was a hydrophilic surface (silicon oxide on prism ITO glass) with square hydrophobic islands (Teflon AF 400s2-100-1, DuPont) of 50 μm with pitch distance of 100 μm, as shown in Fig. 1(a). A typical image of a droplet on a patterned surface is shown in Fig. 1(b). The roughness of the patterned surface is tested by a surface profiler (Dektak 150 Veeco Surface Profiler) and it is shown that the height of Teflon on the substrate is as low as 60 nm and roughness of the patterned surface is small (around 5 nm for Teflon and 25 nm for the substrate, shown as Supplementary Figure S2). This high smoothness is suitable for characterizing the effects of wettability patterns on evaporation of droplets without serious conflating topological effects. Since the Teflon layer is very thin and ITO glass is originally transparent, the microscopic motion of a droplet during evaporation can be clearly revealed and investigated under an inverted optical microscope (Eclipse TE2000-U, Nikon Instruments Inc.), which makes it convenient to investigate the microscopic range contact line movement during evaporation.

The wettability data of various multicomponent droplets on hydrophilic (SiO_2_), hydrophobic (Teflon) and a patterned surface at thermal condition (40 °C) is measured by the method illustrated in Supplementary Figure S1 and calculated by drop shape analysis^40^. As patterns restructured the contact line but the droplets apparently remained symmetric, the focus plane of the camera is adjusted to the cross section of the droplet center which is parallel to the edge of the patterns. In our experiments, the apparent receding and advancing contact angles on the patterned surface were determined by a typical volume change method and the snake-based approach programmed as a plugin for ImageJ, which is a free open-source multi-platform Java image-processing program^40^. According to Stalder *et al*.^40^ the standard deviation for receding angle measurements using the snake-based approach is 0.2 degree. In our experiments, because small deviations in the contact lines may be formed by surface patterns, each contact angle was averaged over 5 measurements. The standard deviation of all measurements is 1.06 degrees given the uncertainty in contact angle measurements is ±2.08 degrees (95% confidence level). The measured receding and advancing contact angles are shown in Table 1. The same method was also applied to recording of the evaporation process on patterned surfaces since the droplet profile is apparently symmetric during the receding process (see Supplementary Figure S1).

The temperature of the substrate is measured by a thermocouple on the top of the surface and the power is adjusted to ensure the surface is stable at 40 °C. Since the thickness of Teflon is only around 60 nm, the temperature difference between the Teflon and silicon oxide surfaces can be negligible. It should be noticed that, since a fluorosilane monolayer was deposited on SiO2 for better adhesion of Teflon, the hydrophobicity was enhanced even though the surface was treated by dry etching.

### Evaporating a multicomponent droplet on a chemical micro-patterned surface

**Figure 2:**
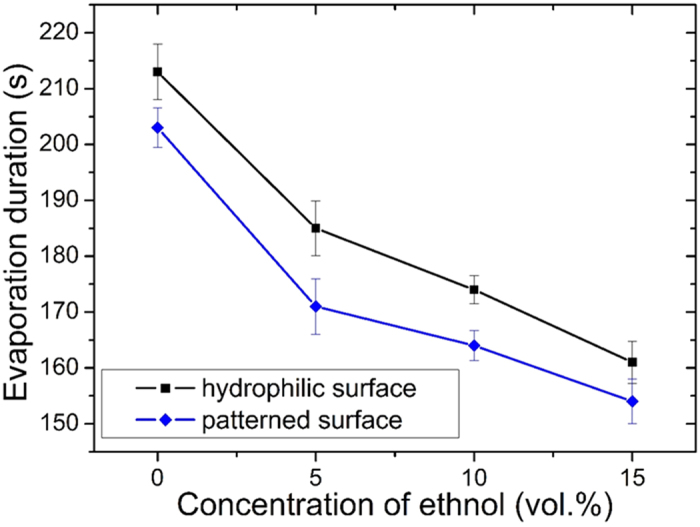
Evaporation duration of droplet with different composition (0 to 15 vol.% ethanol) on homogenous hydrophilic/patterned surface.

To compare the evaporation duration of a multicomponent droplet on a homogenous hydrophilic surface (SiO_2_) and patterned surface, we deposited a multicomponent droplet of 1 μl on both surfaces and recorded the reaction (see Method and Supplementary Figure S1). Results of total evaporation time are shown in Fig. 2. It is found from the results that evaporation of either multicomponent or monocomponent droplets was generally faster on the patterned surface than on the homogenous hydrophilic surface, indicating the patterned surface results in droplet evaporation enhancement which will be discussed in a later section.

**Figure 3:**
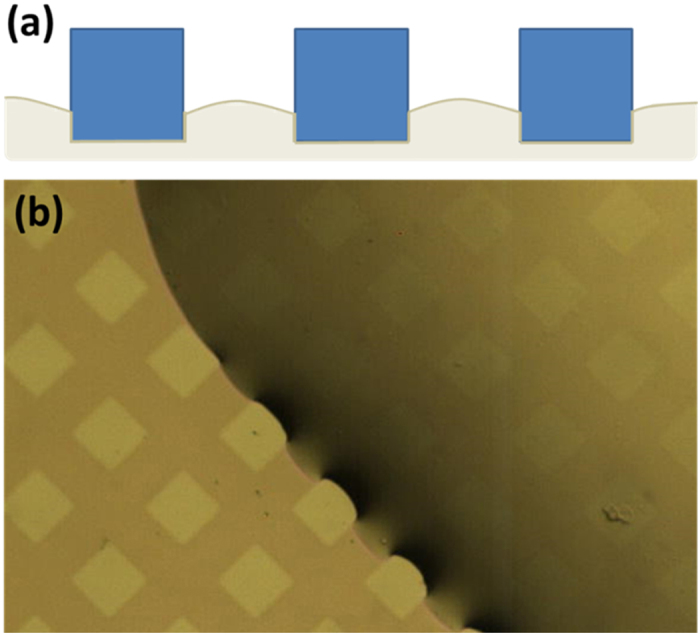
Contact line profile of a multicomponent droplet (15 vol.% ethanol) on patterned surface: (**a**) schematic image of contact line and (**b**) image from microscope.

The enhancement of evaporation introduced by the patterned surface can be attributed to the increase of contact line length due to the hydrophobic square island. Firstly, it is worth mentioning that, for slowly evaporating drops on a hydrophilic surface, the mass flux of vapor from the drop can be described by^41^


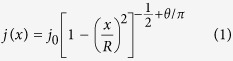


where *θ* (0 < *θ* < *π*/2) is the dynamic contact angle during evaporation, x is the distance from the center of the drop which has radius R, and the prefactor *j*_0_ depends on the saturation pressure, the vapor diffusivity, and the far field concentration. From Eq. 1, the evaporation flux for a droplet is non-uniform and the highest evaporation flux occurs at the contact line, which was confirmed by previous FEM calculation results^10^ and implies the evaporation would be enhanced as the length of the three phase contact line is increased. Figure 3 shows the microscopic contact line profile of a multicomponent droplet (15 vol.% ethanol) on our patterned surface. It is apparent that Teflon islands obstructed the spread of the contact line and the new contact line was created along the islands’ perimeter. Therefore, although the contact area decreases for a droplet on a patterned surface, the total length of the contact line increases, resulting in faster evaporation than with a homogenous hydrophilic surface under identical thermal and composition conditions. The evaporation enhancement is also consistent with Wan *et al*.’s MD (molecular dynamics) simulation results of liquid evaporation on nanoscale hydrophilic-hydrophobic patterned surfaces. To explain this enhancement; as the liquid-air boundary was increased by hydrophobic-hydrophilic patterns, the evaporation of water became faster due to more diffusion out of the water in the hydrophilic region across the liquid-air boundary. Therefore, the longer the contact line is, the faster the droplet evaporates^39^. However, evaporation enhancement in our experiment is only about 5% which is smaller than that of the MD simulation result. In Wang *et al*.’s study, an accelerating region is assumed where evaporated molecules run into an infinite vacuum. The region is 4 nm to 5.5 nm above the surface. Because the water thickness is assumed to be only about 0.5 nm, this assumption will enhance the number of water molecules entering the accelerating region from the surface, i.e, the evaporation rate will be enhanced significantly. However, in our experiments, the droplet thickness is about several hundred microns. The evaporation rate will be significantly reduced due to the thickness of water increased. Therefore, although the mechanism of evaporation rate enhancement may be the same, MD simulation may not be applicable to a mini/micro droplet evaporation condition which causes the difference between the experiments and MD simulations.

### Dynamic behaviors of an evaporating multicomponent droplet on a patterned surface

Besides evaporation enhancement, we also studied and compared the evaporation dynamics parameters of multicomponent droplets on homogenous and patterned surfaces, including contact line movement, contact angle (CA) and base diameter (BD), since they are essential for us to understand the nature of multicomponent droplet evaporation on patterned surfaces.

**Figure 4:**
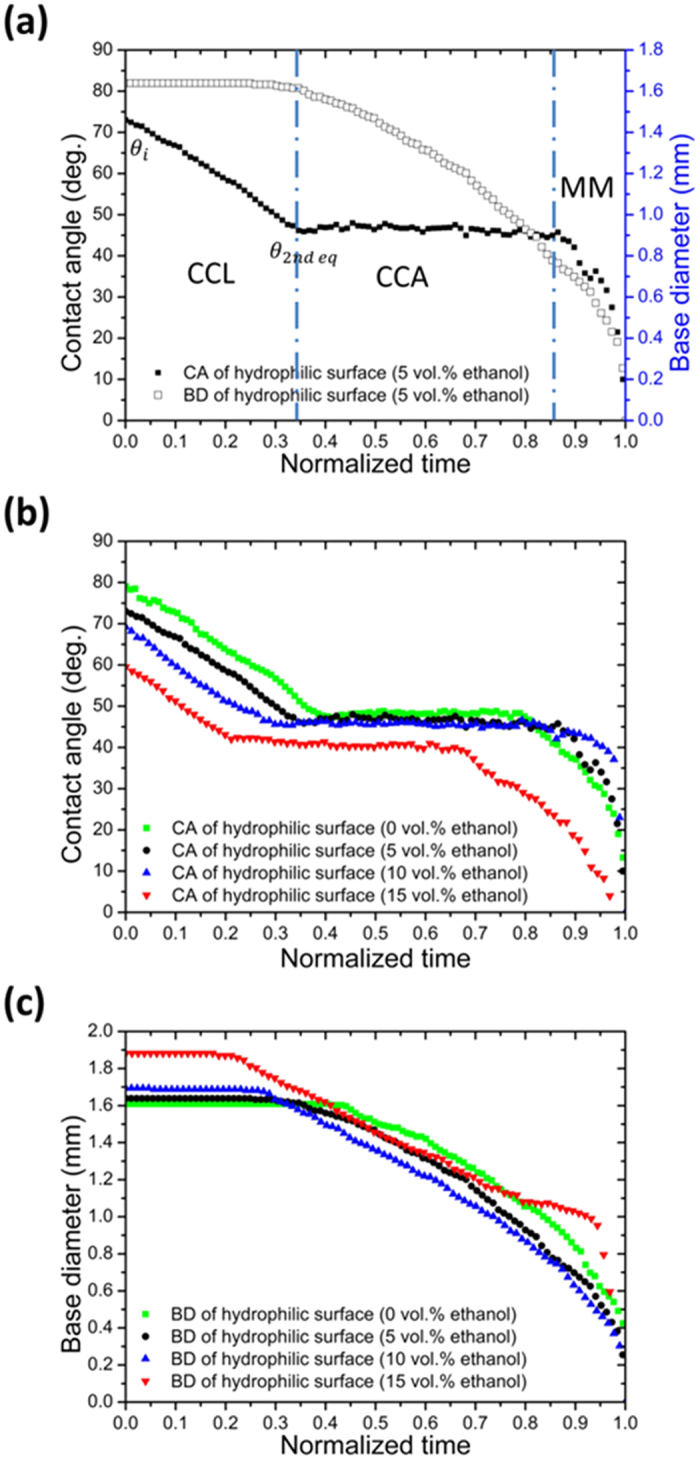
Dynamic evaporation behaviors of multicomponent droplet on homogeneous hydrophilic surface: (**a**) Evolution of the contact angle and of base diameter of an evaporating droplet. (**b**) Typical evolution of contact angle of time for droplets of composition from 0 to 15 vol.% ethanol. (**c**) Typical evolution of base diameter of time for droplets of composition from 0 to 15 vol.% ethanol.

**Figure 5:**
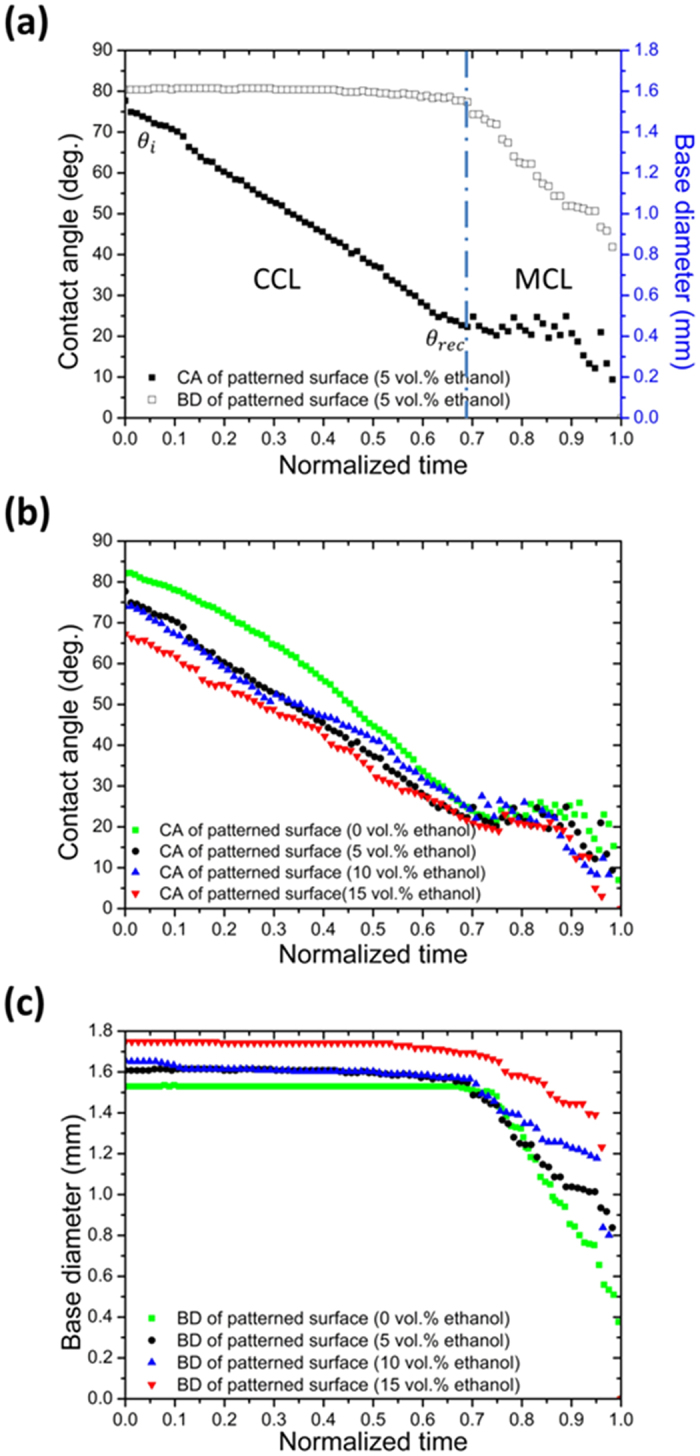
Dynamic evaporation behaviors of multicomponent droplet on patterned surface: (**a**) Evolution of the contact angle and of base diameter of an evaporating droplet; (**b**) Typical evolution of contact angle for droplets of composition from 0 to 15 vol.% ethanol. (**c**) Typical evolution of base diameter for droplets of composition from 0 to 15 vol.% ethanol.

Based on evolution of those dynamics parameters during evaporation, we can easily define different regimes of evaporation processes. Figure 4a shows a typical droplet evaporation on a homogenous surface, which normally includes constant contact line (CCL) regime, constant contact angle (CCA) regime and mix mode (MM), sequentially. In Figs 4 and 5, the normalized time is defined as *t*/*t*_*f*_, where *t* and *t*_*f*_ represent time and the total evaporation duration, respectively. Droplets with all kinds of concentration in our experiments showed similar behavior during evaporation (Fig. 4b and c). As the ethanol concentration increased, the liquid-gas surface tension of the droplet reduces, causing the receding contact angle, also called second equilibrium contact angle *θ*_2*nd eq*_, reduces (shown in Fig. 4b), according to the equilibrium condition of Young’s equation^22^. Since the evaporation rate increases with the ethanol concentration, the receding contact line of multicomponent droplets occurs earlier than monocomponent droplets, shown again in Fig. 4b and c.

However, we found the dynamic evaporation behaviors of a patterned surface are far different from the homogenous hydrophilic surface. Figure 5a shows typical evaporation of a multicomponent droplet on a patterned surface, which obviously exhibits two distinct regimes: (1) droplet of an initial contact angle *θ*_*i*_ begins to evaporate with a constant contact line (CCL) regime; (2) then, the contact line starts to recede when it reaches the critical receding contact angle *θ*_*rec*_ and we determine this regime as the moving contact line (MCL) regime which is characterized by stick—slip behavior of the contact line associated with oscillations of the contact angle. It is observed that similar kinetics occur in all experiments with various compositions, which is reported in Fig. 5b and c.

After comparing measurement results in Figs 4 and 5, it is interesting to find that, while the contact line receded smoothly on the homogenous surface, in the MCL regime of the patterned surface, every contact line movement associated with temporary contact angle changes was stepwise (we define it as ‘jump’). The jump distance is always about 50 um, same as the pattern length. This ‘jump’ is very similar to the one on the micro-structured surface with pillar arrays^42^,^43^, distance of which is close to the interspacing between two neighboring microstructures at the surface. This observation suggests that the stepwise reduction in base diameter is directly related to jumping at the contact line between surface patterns. To further understand this stepwise movement, we investigated the microscopic movement of the contact line in MCL regimes in order to explain the motion and further understand the mechanism under the inverted microscope.

**Figure 6:**
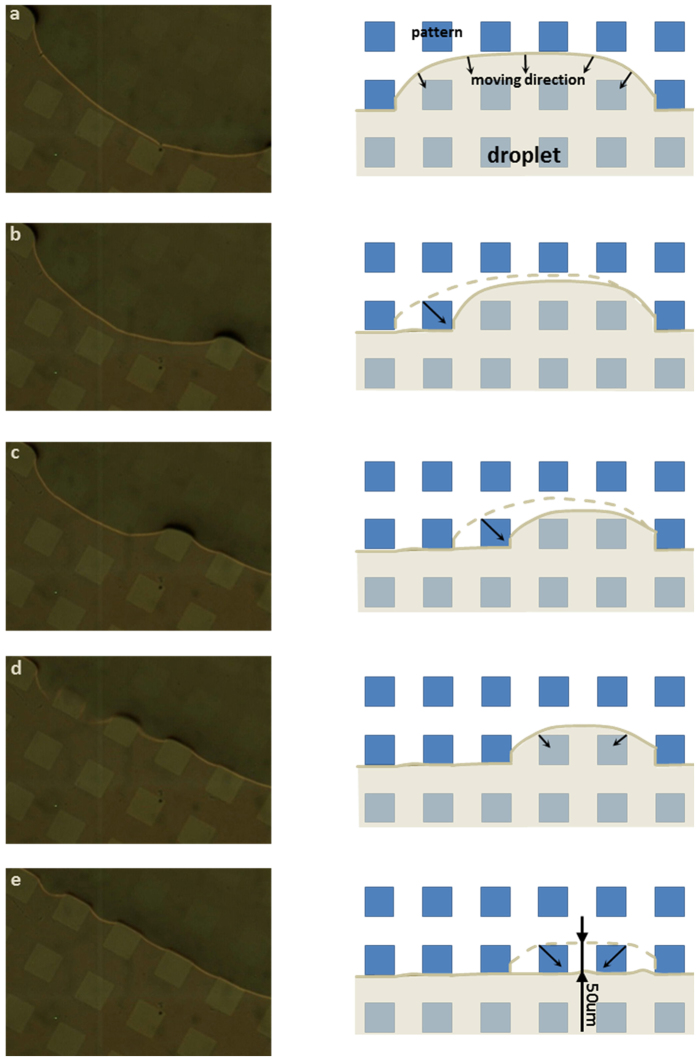
Evolution of the actual contact line of an evaporating droplet (5 vol.% ethanol) on a patterned surface. The microscope images ((**a**–**e**), left) and corresponding schematic images ((**a**–**e**), right) show the sequential depinning progress of the contact line. Dash line and solid line represent the contact line before and after transient movement respectively. The moving direction of the contact line is indicated by arrows.

Figure 6a to e show the evolution of the actual receding droplet contact line during the evaporation and the schematic mechanism (see Supplementary video S4). In the beginning, shown in Fig. 6a, the three-phase contact line was pinned on the hydrophilic area and was distorted by the Teflon islands (going along islands’ boundary). As evaporation continued, in the hydrophilic area, the contact line receded slowly and smoothly, moving in the direction indicated by the arrows in Fig. 6a. In this stage, the contact line movement is similar to the movement on a homogenous surface. However, once it touched the corner of island (Fig. 6b), the contact line detached from the island immediately and then pinned along the island’s boundaries. The contact line repeated this movement (Fig. 6b and c) until the contact line covered only two Teflon islands (Fig. 6d). When the contact line touched the corners of those two islands, the contact line shrank simultaneously from both sides of the islands, forming a straight contact line attached to the base of the Teflon islands (Fig. 6e), which represented the end of receding from that row of Teflon islands and the start of next row. The contact line then continued to shrink from the patterned surface row by row following the mechanism above until the droplet totally evaporated. The microscopic movement we observed in the present work not only explains the mechanism of contact receding and constant ‘jump’ distance which is the same as the pattern length (Fig. 6e), but also provides an insight for controlling mono/multi-component droplet on a patterned surface.

Another interesting finding is that, on a patterned surface, the receding contact angles for droplets of different compositions are very similar (around 21 deg.), reported in Table 2, while the receding contact angles for droplets on a homogenous surface vary with ethanol concentration apparently (from 49.1 to 42.8 deg. for 0 to 15 vol.%).

Here, we employ a local force model as an analytical tool to explain why critical contact angles show a large difference on homogeneous and chemical micro-patterned surface. For a homogeneous surface, a moving contact line could be explained by the local forces at the contact line, including the depinning and pinning forces which we apply to our analysis in the following section.

The depinning force *F*_*d*_ which leads to contact line detachment can be described as^43^,^44^,^45^


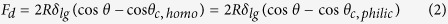


where *R* is the base radius of droplet, *δ*_*lg*_ is the surface tension of water, *θ* is the contact angle during the evaporation, and *θ*_*c,homo*_ is the apparent contact angle on the homogenous surface. In our case the homogenous surface is a hydrophilic surface. The pinning force, which keeps the contact line still, can be expressed as


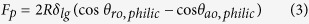


where *θ*_*ro,philic*_ and *θ*_*ao,philic*_ are receding and advancing contact angles on the homogeneous hydrophilic surface. When the driving ratio,


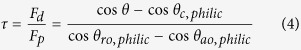


is equal to 1, the contact line will begin to recede and the droplet will enter CCA regime from CCL regime. To verify this model, the critical receding contact angle of a monocomponent droplet (0 vol.% ethanol concentration) is calculated from *τ* = 1, using the data of Table 1, and the result is cos *θ*_*rec*_ = 0.643 and *θ*_*rec*_ = 50.0°. The calculated receding contact angle is close to the actual value, which proves the model above is feasible.

From the data of Table 1, we can see that the depinning term, cos *θ*_*ro,philic*_−cos *θ*_*ao,philic*_, increases with ethanol concentration from 0.445 to 0.514 of 0 to 15 vol.% ethanol concentration. That means the value of cos *θ* should be larger when ethanol concentration increases to let τ reach 1, which is consistent with our measurement results where the initial receding contact angle drops from 49.1° to 42.4° of 0 to 15 vol.% ethanol concentration. However, only qualitative conclusion can be drawn for a multicomponent droplet because the exact concentration of a multicomponent droplet at every moment during evaporation is unknown. But this model still gave us a good explanation about the receding contact angle of the drops with ethanol concentration.

Now we consider the critical receding contact angles of multicomponent droplets on a patterned surface, which are shown to be similar with each other. First, recalling our previous study of multicomponent droplet evaporation on a hydrophobic surface^21^, the ethanol finished evaporation before 30% of the whole evaporation duration, which is indicated by the internal pattern change from transition to upward flow pattern. This previous result implied that, as the receding occurred at about 75% of whole evaporation, the droplet became a pure water droplet at that time and that is why the receding contact angles are similar. Although this guessing sounds reasonable, the condition in this study is not the same as before, especially the patterned surface. Therefore, a solid explanation is needed and further analysis should be carried out.

In some previous studies about droplet evaporation on a micro-pillar surface, the initiation of moving the contact line was also successfully explained considering the local forces at the contact line, similar to the analysis for a homogenous surface^43^,^46^, and thus we apply it to our investigation again. Similar to a homogenous surface, the depinning force *F*_*d*_ can be described as


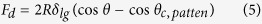


where *R* is the base radius of the droplet, *δ*_*lg*_ is the surface tension of water, *θ* is the contact angle during evaporation, and *θ*_*c,pattern*_ is the apparent contact angle on the patterned surface. Different to the homogeneous surface, the expression for the pinning force is complex, which for a pillar surface in refs 43, 46 and 47 can be expressed as^48^


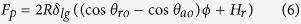


where *θ*_*ro*_ and *θ*_*ao*_ are receding and advancing contact angles on the flat surface, *ϕ* is the hydrophilic area fraction of the total surface area. *H*_*r*_ is proportional to *D*^2^/*L*^2^, where *D* and *L* represent the pattern length and pattern-to-pattern spacing, denoting the adhesive force due to microscale surface roughness and heterogeneity. However, for our case which is not 3D microstructure surface but chemical micro-patterned surface, Eq. 3 is improved according to our condition as follows:


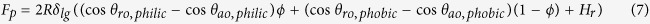


where *θ*_*ro philic*_ and *θ*_*ao philic*_ are receding and advancing contact angles on the hydrophilic surface as mentioned above, *θ*_*ro,phobic*_ and *θ*_*ao,phobic*_ are receding and advancing contact angles on the hydrophobic surface, *ϕ* is the hydrophilic area fraction, 
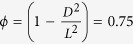
 and *H*_*r*_ equals to 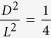. Then the driving ratio could be expressed as


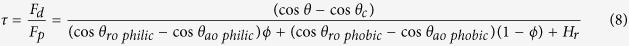


As mentioned above, when τ is equal to 1, the contact line will begin to recede and the droplet will turn from CCL regime to MCL regime. Setting ethanol concentration identical to the initial state during evaporation and τ = 1, we can calculate the critical receding contact angle *θ*_*rec*_ for different multicomponent droplets with data from Table 1.


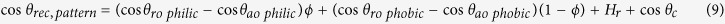


**Figure 7:**
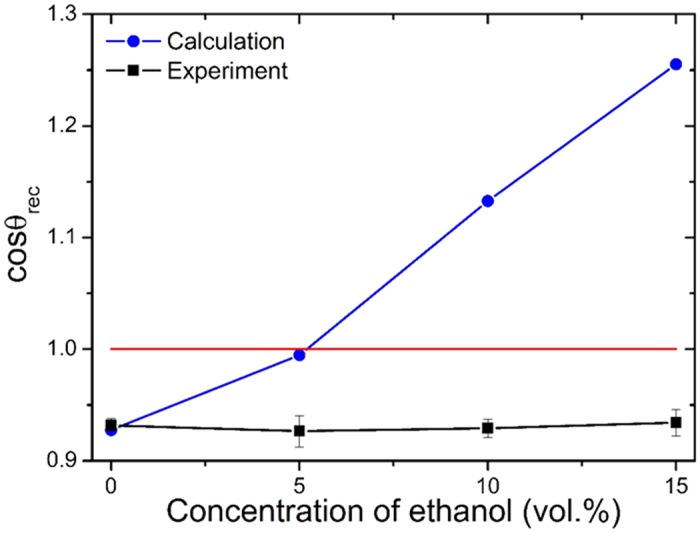
Calculation results of cosine value of critical receding contact angle (*θ*_*rec*_) of various multicomponent droplets when driving ratio *τ* = 1, assuming no change of composition.

From the results reported in Fig. 7, we find that cos *θ*_*rec,pattern*_ value increases with ethanol concentration, which means it is hard for contact line to detach in high ethanol concentration while the critical receding contact angle decreases with ethanol concentration. The cos *θ*_*rec*_ of 5 vol.% ethanol leading to MCL regime is 0.994, corresponding *θ*_*rec*_ = 6.4° which is far below the actual critical receding contact angle. Values of cos *θ*_*rec*_ of 10 and 15 vol.% ethanol droplets are even larger than 1, suggesting the contact line would never recede. However, the result of water (0 vol.% ethanol) fits the experiment results well: cos *θ*_*rec,pattern*_ of water is 0.923, corresponding *θ*_*rec,pattern*_ = 22.6°, which is not only very close to the actual receding contact angle of 0 vol.% ethanol droplet but also those of 5 to 15 vol.%.

Hence, we can conclude that, when the contact line began to recede, droplets mainly contained water without ethanol and thus the receding contact angles are similar for different compositions in our tests, which is consistent with our assumption above This conclusion proved that a chemical micro-patterned surface can not only control the multicomponent droplet dynamic behavior but can also be applied to predict when the volatile composition of a multicomponent droplet completes evaporation, which is beneficial for many industrial applications. In addition, this improved local force model gives us a new understand of receding conditions for multicomponent droplets and can be used as a good tool to predict droplet behavior on a chemical micro-patterned surface.

## Conclusions

In present work, we report the finding of chemical pattern surface effects on an evaporating multicomponent droplet. The influence of the patterned surface on the duration of droplet evaporation can be shortened and the evaporation is enhanced significantly. This enhancement resulted from strengthened diffusion out of the hydrophilic region across the contact lines as the contact line is elongated by the patterned surface.

Regarding the dynamic behavior, it was found that the evaporation process of droplets on a patterned surface can be mainly divided into two regimes, constant contact line (CCL) regime and moving contact line (MCL) regime, which are different to the three regimes for a homogenous surface. Taking advantage of the transparent substrate, the mechanism of microscopic motions of the contact line in MCL regime was explored using an inverted microscope. The contact line receded pattern by pattern on the same row of patterns until it covered only two Teflon islands. Then the contact line shrank simultaneously from both sides of the two Teflon islands and began receding from the next row of patterns. This mechanism explained the stepwise motion of the contact line and similarity of jump distance to pattern length.

In addition, it was interesting to find that the critical receding contact angles of different multicomponent droplets, which indicated transition from CCL to MCL regime, were closed, while critical receding contact angles varied on the homogenous surface. To understand those phenomena, an analysis based on improved local forces at the contact line was utilized on both the homogenous and patterned surface, including pinning and depinning forces. Our local force model was in good agreement for the critical receding contact angle and can be used as a tool to predict droplet behavior on a chemical micro-patterned surface. It is also confirmed that droplets were monocomponent (water) when the MCL regime started, which implies that a chemical micro-patterned surface can be applied to predict when the volatile composition of a multicomponent droplet completes evaporation.

In summary, the findings of the present work give a better understanding of multicomponent droplet evaporation on a wettability-patterned surface and mechanism of dynamic evaporation behavior. Those findings might also provide important insights for the design of structured surfaces, coupled with the capability to control evaporation behavior and evaporation rate, for various applications especially for multicomponent droplets.

## Method

### Experimental setup

The experimental setup for droplet evaporation experiments is shown in Supplementary Figure S1. To investigate the evaporation characteristic, including dynamic contact angle, base diameter and evaporation duration, of a multicomponent droplet on a patterned surface, CCD camera (MotionXtra HG-100K, Redlake Ltd.) with a long distance zoom lens (Zoom 6000, Navitar Inc.) was applied to record the whole evaporation process. Since our substrate is transparent, an inverted microscope (Eclipse TE2000-U, Nikon Instruments Inc.) was also applied in the experiments in order to reveal the microscopic motion of the contact line and investigate the dynamic evaporation behavior during multicomponent droplet evaporation. Multicomponent droplets with a volume of 1 ± 0.05 μl were generated by a Hamilton micro syringe (Hamilton, 5 μl TLC Syringes with removable needle of gauge 33, 210 um of outside diameter) and gently deposited on the heated homogeneous/patterned surface with constant substrate surface temperature T_sub_ = 40 °C. Each droplet was deposited in the same place to ensure reproducibility. To avoid placing the droplets on the wetted surface, after each experiment, the substrate was cleaned with ethanol and DI water, dried with compressed air and then kept in an oven at 105 °C for 15 min in order to totally dry. The liquid for the tested samples was deionized water based mixtures with ethanol, in which the concentration varied from 0% to 15% by ethanol vol.%. The mixtures for experiments were freshly made before every test and the accuracy of concentration is ±0.05 vol.%. During all experiments, the local surroundings were kept at 296 ± 1 K (23 ± 1 °C) and 50 ± 3% RH and each experiment for droplet evaporation was carried out for more than five runs to identify the reproducibility.

### Sample fabrication process

The prism substrate is transparent glass coated with an ITO layer of 100 nm thickness as a resistive heater. After depositing two gold electrodes of 200 nm thickness, a silicon dioxide passivation layer of 500 nm was deposited on the ITO layer on which the patterned surface was fabricated. Supplementary Figure S3 shows the image of the patterned surface and the fabrication process. The details of the fabrication procedure are described as follows:

1. Teflon layer of 60 nm thickness was deposited on the substrate by spin coating. Following suggestions by Dupont, a layer of fluorosilane (1H,1H,2H,2H-Perfluorodecyltriethoxysilane, Sigma-Aldrich Co. LLC.) was deposited to improve the adhesion of Teflon on the silicon oxide before spin coating.
2. To pattern the Teflon, standard photolithography technique was utilized with help of alumina layer by ALD (atomic layer deposition), as it is very difficult for photoresist to directly adhere to a Teflon surface.
3. To realize the patterned surface, unwanted Teflon and alumina was removed by dry etching, then residual photoresist and alumina was also removed by MS-2001 photoresist stripper (Fujifilm Electronic Materials Co., Ltd) and NaOH solution (concentration of 5 mol/L), respectively.

## Additional Information

**How to cite this article:** He, M. *et al*. Multicomponent Droplet Evaporation on Chemical Micro-Patterned Surfaces. *Sci. Rep.* **7**, 41897; doi: 10.1038/srep41897 (2017).

**Publisher's note:** Springer Nature remains neutral with regard to jurisdictional claims in published maps and institutional affiliations.

## Supplementary Material

Supplementary Information

Supplementary Video S4

Supplementary Video S5

## Figures and Tables

**Table 1:** Wettability data of patterned surface at 40 deg. C.

Ethanol concentration (vol.%)	Hydrophilic surface			Hydrophobic surface		Patterned surface
	Receding contact angle	Advancing contact angle	Initial contact angle	Receding contact angle	Advancing contact angle	Initial contact angle
0	62.1	88.7	78.6	84.5	134.9	82.1
5	54.6	82.5	75.0	78.3	130.5	78.5
10	45.7	75.8	69.3	67.5	127.2	73.4
15	33.2	71.2	59.5	61.1	124.1	69.0

**Table 2:** Receding contact angle of multicomponent droplets with different composition on homogenous and patterned surfaces (data averaged from five experiment results).

Ethanol concentration (vol.%)	Critical receding contact angle of homogenous surface (deg.)	Critical receding contact angle of patterned surface (deg.)
0	49.1	21.7
5	47.3	22.1
10	46.5	22.3
15	42.8	20.9
