# Supplementary material for: Multicomponent Droplet Evaporation on Chemical Micro-Patterned Surfaces
Source: Sci Rep. 2017 Feb 3;7:41897. doi: 10.1038/srep41897 (PMC5291323; doi:10.1038/srep41897)
Supplement: Supplementary Information [file srep41897-s1.pdf]

Supplementary documents

Evaporative Characteristic of Multicomponent Droplets on Chemical Micro-Patterned Surfaces

Minghao HE, Dong LIAO and Huihe QIU*

Department of Mechanical and Aerospace Engineering, The Hong Kong University of Science and Technology, Clear Water Bay, Kowloon, Hong Kong SAR, China

Correspondence and requests for materials should be addressed to Huihe Qiu (e-mail: meqiu@ust.hk)

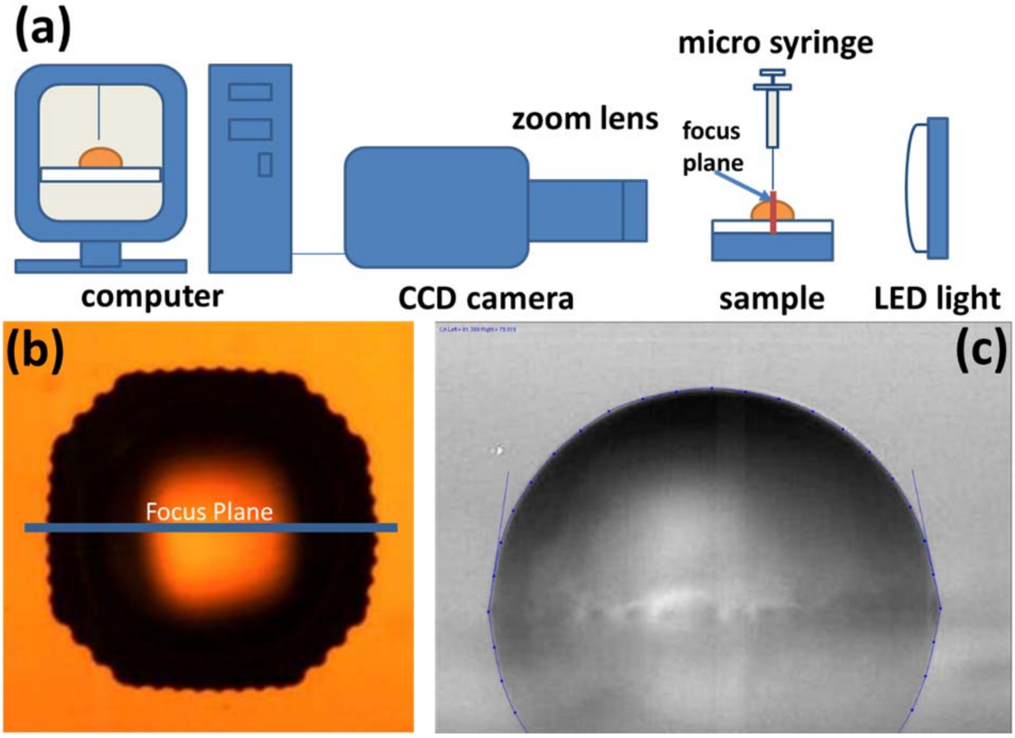

S1: Experimental setup and contact angle measurement method: (a) schematic of experimental setup for droplet evaporation; (b) bottom view of the droplet on patterned surface and corresponding focus plane of contact angle measurement; (c) contact angle measurement result by snake-based approach of Image J[40].

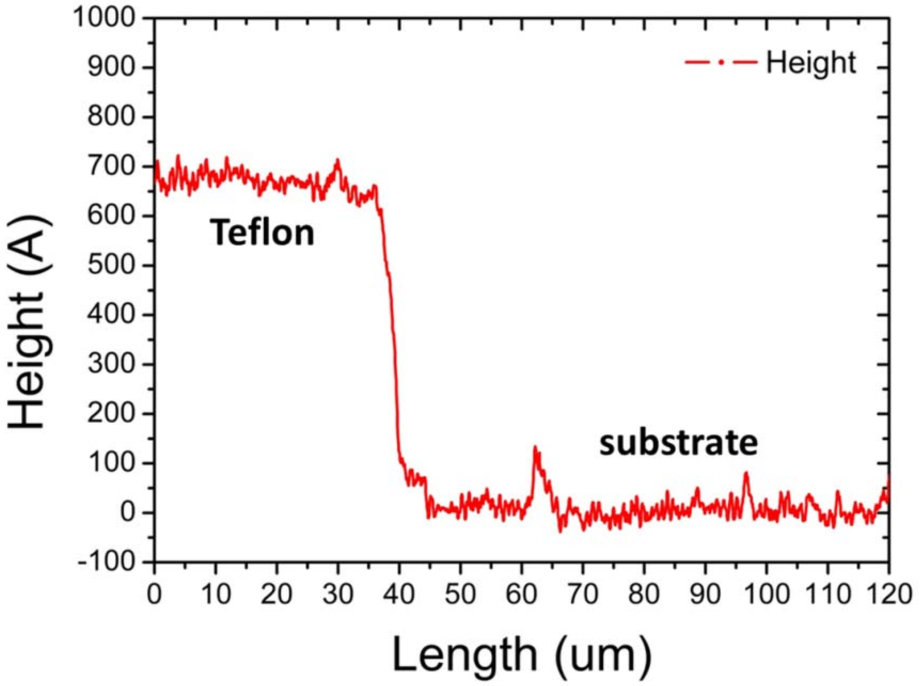

S2: Profile of patterned surface: height of Teflon is about 60 nm and the roughness is around 5 nm for Teflon and 25 nm for substrate.

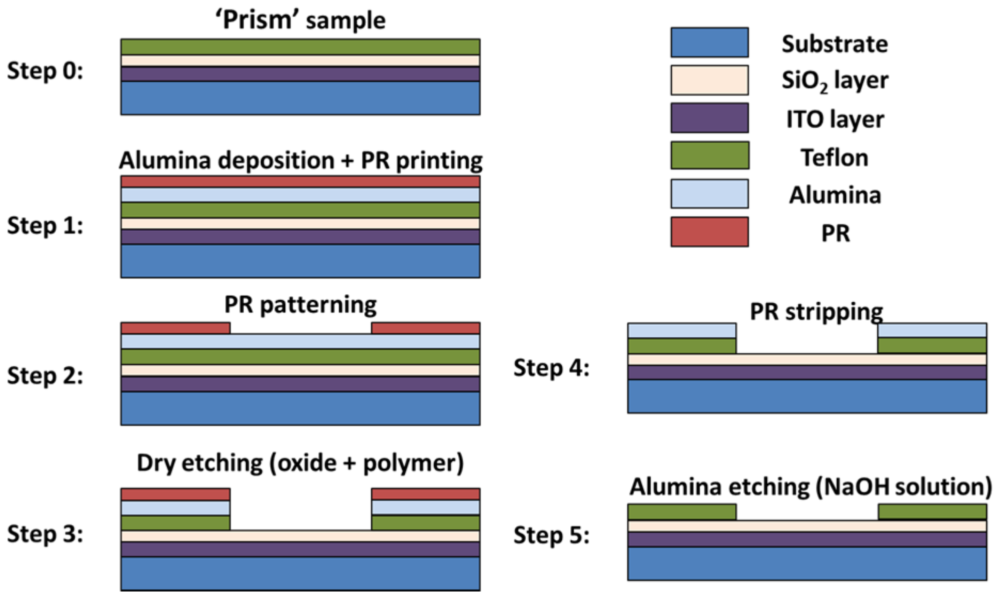

S3: Fabrication procedure of patterned surface

S4: The contact line movement during the evaporation (Right hand side view)

S5: The contact line movement during the evaporation (Left hand side view)
